# The Effect of Dietary Supplementations on Delaying the Progression of Age-Related Macular Degeneration: A Systematic Review and Meta-Analysis

**DOI:** 10.3390/nu14204273

**Published:** 2022-10-13

**Authors:** Susanne Csader, Sonja Korhonen, Kai Kaarniranta, Ursula Schwab

**Affiliations:** 1 School of Medicine, Institute of Public Health and Clinical Nutrition, University of Eastern Finland, 70211 Kuopio, Finland; 2 School of Pharmacy, Faculty of Health Sciences, University of Eastern Finland, 70211 Kuopio, Finland; 3 School of Medicine, Institute of Clinical Medicine, Department of Ophthalmology, University of Eastern Finland, 70211 Kuopio, Finland; 4 Department of Ophthalmology, Kuopio University Hospital, 70211 Kuopio, Finland; 5 Department of Medicine, Endocrinology and Clinical Nutrition, Kuopio University Hospital, 70211 Kuopio, Finland

**Keywords:** age-related macular degeneration, omega-3 fatty acids, supplements, carotenoids, meta-analysis, xanthophylls

## Abstract

*Purpose*: Age-related macular degeneration (AMD) is a neurodegenerative ophthalmic disease. The purpose of this systematic review (SR) and meta-analysis was to evaluate if dietary supplementation alone or in combinations might delay the progression of any of the stages of AMD. *Methods:* A SR and meta-analysis identifying cohort studies and randomized controlled trials (RCTs) evaluating the effect of supplements in patients diagnosed with AMD. PubMed, Scopus, Web of Science, CINAHL, and Cochrane were searched through 8th October 2021. *Results:* Twenty studies, examining 5634 participants ranging from 55 to 80 years, were included in the SR. Eight studies were selected for meta-analysis (414 and 216 subjects in the intervention and control groups). Lutein and zeaxanthin plus *n*-3 long-chain polyunsaturated fatty acids (*n*-3 LC-PUFA) supplementation showed significant improvements in best-corrected visual acuity (BCVA) (SMD: −1.99, 95% CI: −3.33, −0.65) compared to the control group. Multifocal electroretinogram results (mfERG) were significantly improved overall (SMD: 4.59, 95% CI: 1.75, 7.43) after lutein plus zeaxanthin supplementation. *Conclusions:* Combinations of lutein and zeaxanthin with *n*-3 LC-PUFA might be beneficial in preventing AMD progression and deterioration of visual function. Our results encourage initiating further studies with combinations of *n*-3 LC-PUFA, lutein, and zeaxanthin especially in early AMD patients.

## 1. Introduction

Age-related macular-degeneration (AMD), a multifactorial neurodegenerative ophthalmic disease, is the most common cause of visual loss in the elderly population in the industrialized countries [1]. Nowadays, around 200 million people are affected by AMD, and about 9% of these cases terminate in total blindness [2]. The prevalence of AMD is expected to increase up to 300 million people by 2040, which will pose a major burden for the public health system as well as reducing the quality of life for the affected individuals [3].

AMD is subdivided into dry (80–85% of cases) and wet AMD (10–15% of cases) forms. AMD is a complex disease involving increased oxidative stress, protein aggregation, inflammation, and in wet AMD cases, angiogenic processes [4]. The hallmarks of the different AMD stages include pigment mottling in retinal pigment epithelial (RPE) cells [4], the presence of intracellular lipofuscin and the formation of extracellular drusen [5], and finally, retinal atrophy. In wet AMD, retinal edema, lipid exudates, and hemorrhages are usually observed. The principal non-genetic risk factors of AMD are age, hypertension, hypercholesterolemia, smoking, physical inactivity, obesity, and a low dietary intake of antioxidants [6]. The main genetic variants associated with AMD are complement factors H and I (*CFH*, *CFI*), complement components *C2* and *C3*, and age-related maculopathy sensitivity 2 (*ARMS2*) [7]. In addition to the classification into dry and wet AMD, the disease is subdivided into early, intermediate and late AMD stages depending on the amount and size of drusen, pigmentary abnormalities, retinal thickness, and the macular neovascularization (MNV) state [2,6,8]. While most people are asymptomatic in early AMD, intermediate macular degeneration can cause mild distorted vision and/or decreased color and contrast sensitivity (CS) [2,9]. Late AMD, subdivided into geographic atrophic (GA) and MNV, is usually associated with a clear visual decline and even blindness.

Currently, no pharmacological treatment exists to cure the more prevalent dry AMD form [10], while wet AMD is treated with intravitreal anti-vascular endothelial growth factor (anti-VEGF) inhibitors [11,12]. Repeated injections and high drug costs represent a heavy load on the treating ophthalmology clinics, and the frequent drug administrations are not patient-friendly. Therefore, there is an urgent need for new treatment options. Dietary supplementations would represent a non-invasive and cost-effective option which would be expected to be highly patient-friendly and thus associated with good compliance. Several investigators have reported improvements in AMD progression using different supplement approaches. Furthermore, the Age-Related Eye Disease Study (AREDS) conducted between 1992 and 2001 showed that dietary supplements containing high levels of antioxidants and zinc could delay the progression of intermediate AMD to late AMD and vision loss (25% and 19% risk reduction as compared to placebo, respectively) [13]. From 2006 to 2012, AREDS was followed by AREDS2, which enrolled only patients with intermediate AMD [14]. AREDS2 showed that enhancing the original AREDS formulation with *n*-3 long-chain polyunsaturated fatty acids (*n*-3 LC-PUFA) or lutein combined with zeaxanthin did not confer an additional overall benefit on the risk of progression to late AMD. However, the administration of antioxidants along with lutein and zeaxanthin led to an incremental beneficial increase as compared to patients taking the original AREDS formula. In addition, the population in AREDS2 consisted only of patients with intermediate AMD which limits the trial’s applicability to other major AMD patient groups. Nonetheless, the outcomes of the two AREDS trials demonstrate that there is a clear potential for developing novel nutritional supplement formulations for delaying the progression of different AMD stages.

Furthermore, there is evidence that patients carrying different risk alleles for AMD might benefit from personalized nutritional supplementation [7]. However, there is very limited current knowledge of how the genetics and different nutritional supplements affect the progression of AMD. Thus, acquiring information of the most effective nutritional supplements is crucial before embarking on further studies with dietary supplements i.e., trials in carefully classified AMD patient subpopulations while taking into account genetic information.

Until today, most of the systematic evidence has focused on the prevention of AMD with various nutritional supplementations. However, the effect of nutritional supplementations on delaying the progression of AMD has mostly been overlooked. Thus, this systematic review (SR) with meta-analyses aimed to show if dietary supplementation is beneficial and if so, which of these options, such as carotenoids, *n*-3 LC-PUFA, and vitamins alone or in combinations, might delay or even improve the progression of any AMD stages.

## 2. Materials and Methods

The SR was conducted according to the Preferred Reporting Items of Systematic Reviews and Meta-analysis (PRISMA) statement guideline and the Patient, Intervention, Comparison, and Outcome statement (PICO) [15,16]. In addition, the protocol of this SR was registered in the PROSPERO database (CRD42021290620).

### 2.1. Search Strategy and Data Sources

The authors undertook a systematic literature search in PubMed, Scopus, Web of Science, CINAHL, and Cochrane together with an information specialist to compile all of the cohort studies and randomized clinical trials (RCTs) which have examined the effect of supplements in patients diagnosed with AMD. An extensive search of the PubMed, Scopus, Web of Science, and Cochrane databases was first performed until 8 October 2021, using keywords such as “macular degeneration”, “geographic atrophy”, “dietary supplements”, “vitamins”, “antioxidants”, “cohort studies”, and “randomized controlled trial”. The exact search strings can be found in Supplementary File S1.

### 2.2. Eligibility Criteria: Inclusion and Exclusion

Studies with the following conditions were included in the SR: (1) Cohort studies or randomized control trials in the adult population (>18 years) written in English; (2) diagnosed AMD subjects, including early or intermediate dry AMD, exudative AMD, and geographic atrophy; (3) dietary supplement intervention; (4) control group without supplements or placebo, and (5) an intervention period of at least six months.

The exclusion criteria were (1) uncontrolled studies, (2) interventions with food groups, and (3) animal studies, review articles, case reports, conference abstracts, and trial entries.

### 2.3. Screening, Data Extraction, and Quality Assessment

The list of articles was independently screened by Susanne Csader (S.C.) and Sonja Korhonen (S.K.) based on the abstract and title using RAYYAN [17]. Duplicates were removed, and all studies that did not fit into the above-mentioned inclusion and exclusion criteria were excluded. The remaining full-text articles were independently reassessed by the same authors. In case of a disagreement, Ursula Schwab (U.S.) was consulted. After defining the included studies, the following outcome variables were extracted: (1) best-corrected visual acuity (BCVA), (2) macular pigment optical density (MPOD), (3) multifocal electroretinogram (mfERG), (4) contrast sensitivity (CS) and (5) optical coherence tomography (OCT).

A quality assessment was performed independently by S.C. and S.K. via the Cochrane risk-of-bias tool for randomized trials (RoB)2 tool (version August 2019) for all RCTs and Robins-I (version 2016) for non-randomized trials [18,19].

### 2.4. Data Preparation

Values of BCVA presented in Early Treatment Diabetic Retinopathy Study (ETDRs) letter were converted to logMAR according to the formula: *logMAR = −0.02 × ETDRS + 1.7* [20].

In one study, results of the left eye group and right eye group were combined according to the Cochrane Handbook for Systematic Reviews of Interventions [21,22]. Outcome data from two studies were extracted from figures using the WebPlotDigitizer (https://automeris.io/WebPlotDigitizer/ (accessed on 6 March 2022) [23,24]. After that, changes in means and standard deviation (SD) before and after the intervention were calculated for each parameter according to the Cochrane Handbook [21].

### 2.5. Statistical Analyses

All meta-analyses were conducted using R programming software (version 4.1.1) and the packages “meta” and “metafor”. The outcome variables BCVA and mfERG were continuous, and therefore the standardized mean difference (SMD) was used to measure the effect size and presented as a 95% confidence interval (CI) of the SMD. Random-effects or fixed-effect models were used to calculate and pool the SMDs of all studies from baseline to the endpoint between groups (intervention vs. control). *I*^2^ was used to test the heterogeneity across the studies. A random-effects model was utilized to estimate the mean of the distribution of effects since the studies varied [25].

The analyzed results of supplement effects on BCVA scores and mfERG responses are shown in forest plots. Subgroup analyses were performed by combining different outcome measurements with distinct supplement combinations. In addition, mfERG responses were also divided according to the areas analyzed, i.e., ring 1 and ring 2, representing the sensitivity of different parts of the retina.

Publication-bias analysis such as funnel plot analysis could not be performed due to the lack of statistical power.

## 3. Results

### 3.1. Search Results and Study Characteristics

**Figure 1:**
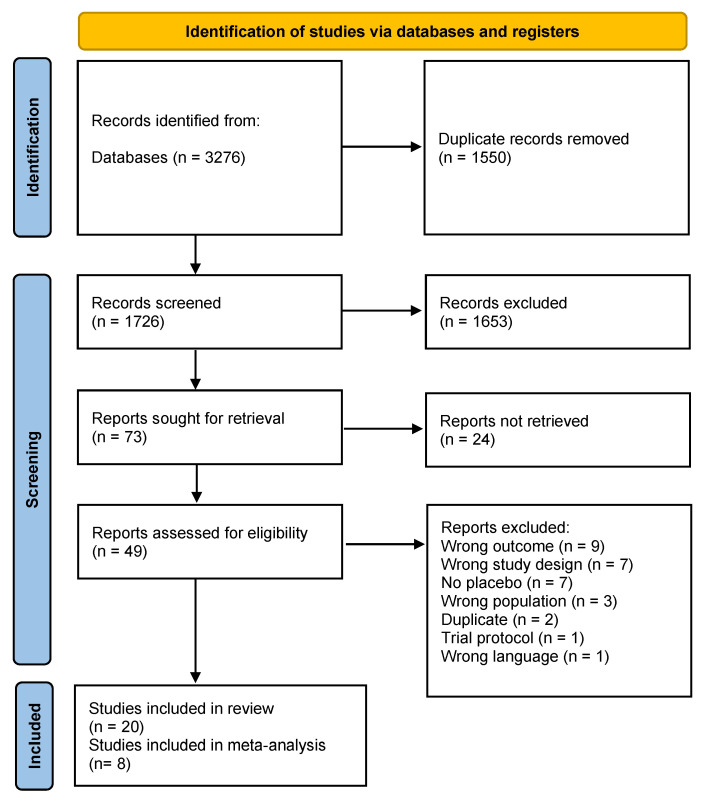
Prisma study flow diagram 2020.

The search yielded 3276 potential records. After removing 1550 duplicates, 73 studies were retained for full-text assessment after screening the studies based on title and abstract. In accordance with the inclusion and exclusion criteria, 20 studies were included for the SR, and 8 of them were considered appropriate for meta-analysis (Figure 1). Two studies had two intervention arms [22,26] and two studies had three intervention arms [13,27]. Huang et al. is the same study as that of Ma et al. but includes other outcomes and a longer follow-up [23]. One study had two control arms with placebo-treated AMD and healthy individuals, respectively [24]. If studies included in the meta-analysis had more than one intervention arm, each of the intervention arms was counted as a separate RCT [26,27]. In the trial conducted by Parisi et al., the placebo treated AMD group was considered as the control group [24]. In total, these 20 studies examined a total of 5634 participants with a mean age ranging from over 55 to 80 years. Out of this, 414 and 216 subjects in the intervention and control groups were included in the meta-analyses. The sample sizes of all of the included studies varied from 14 to 3610 [13,28] and for the meta-analysis from 44 to 145 [26,29]. Two studies provided no data about the gender distribution [28,30] and three studies pooled the gender distribution for the intervention and control group [26,31,32]. Four studies utilized no placebo supplements [24,33,34,35]. The disease stages ranged from early AMD up to wet AMD. The outcome measurements varied extensively between the studies (Table 1) but the most common were BCVA, MPOD, CS, and mfERG.

### 3.2. Quality Assessment

**Figure 2:**
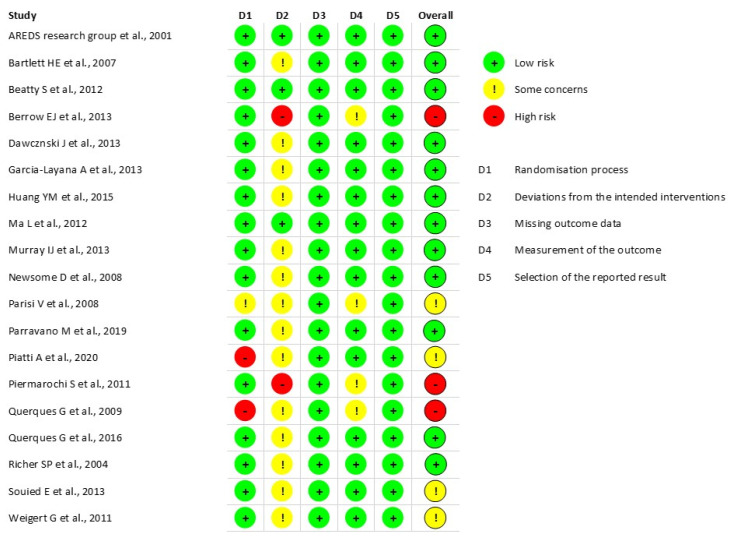
Quality assessment of all randomized controlled trials [13,22,23,24,26,27,28,29,30,31,32,34,35,36,37,38,39,40,41].

Allegrini et al. was the only non-RCT assessed with the Robins-I tool [33]. This study had a low risk of bias. For the 18 RCTs assessed by the ROB2-tool (Figure 2), 12 studies had an overall low risk of bias, there were some concerns about four, and three had an overall high risk of bias. Most issues occurred during the deviations from the intended interventions. The appropriate analysis used to estimate the effect of the intervention assignment had not been mentioned, or a per-protocol had been applied. In some studies, the randomization process revealed some risks due to a lack of detailed use of a random allocation sequence and some differences existed between the groups already at baseline. Four studies had some concerns in measuring the outcome because of the possible influence of the assessor during outcome measurements.

### 3.3. Dietary Supplements Reports and Meta-Analysis

Our aim was to investigate if dietary supplements could halt the development of advanced GA or wet AMD from early or intermediate dry AMD. These are major changes in ocular health and are assessed with different clinical approaches such as BCVA, mfERG, MOPD and CS. Furthermore, a change in the amount of anti-VEGF injections in wet AMD was also defined as an outcome measurement. However, with the limited number of publications, the diverse supplement combinations and varied outcome measurements, we were not able to utilize all of the gathered data in our meta-analysis. In addition, as our study aimed to produce results that could be clinically applicable and relevant, after consulting with an ocular physician, K.K, we focused on those methods that are regularly used in the clinics, such as BCVA and mfERG.

The most extensively used supplement compounds were two xanthophylls of the carotenoid group i.e., lutein and zeaxanthin. Lutein alone had been examined in six studies [22,23,27,31,37], combined with zeaxanthin in six [23,24,27,30,34,38], and combined zeaxanthin and *n*-3 LC-PUFA in four trials [26,28,29,39]. Three studies used *n*-3 long-chain polyunsaturated fatty acids (*n*-3 LC-PUFA) (docosahexaenoic acid (DHA) and eicosapentaenoic acid (EPA)) [32,35,40]. Zinc was administered in two studies [13,41] and curcumin in one study [33]. Several studies utilized supplement complexes such as Macuprev^®^, Ocuvite Duo^®^, FloraGlo^®^, and OcuPower^®^ which included antioxidants and vitamins (Table 1). A detailed list of all ingredients in the complex can be found in the Supplementary File S2.

Eight studies were found to be suitable for meta-analysis based on the available data, common outcome measurements, and common supplement interventions. All eight studies used carotenoid supplements; these have been divided here into three meta-analyses.

### 3.4. Lutein and Zeaxanthin

**Figure 3:**
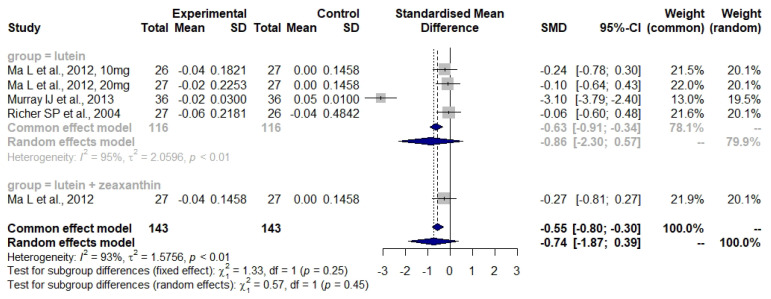
Forest plot of BCVA values in studies where subjects were administered either lutein or lutein plus zeaxanthin; SD, standard deviation; SMD, standardized mean difference; CI confidence interval [22,27,37].

**Figure 4:**
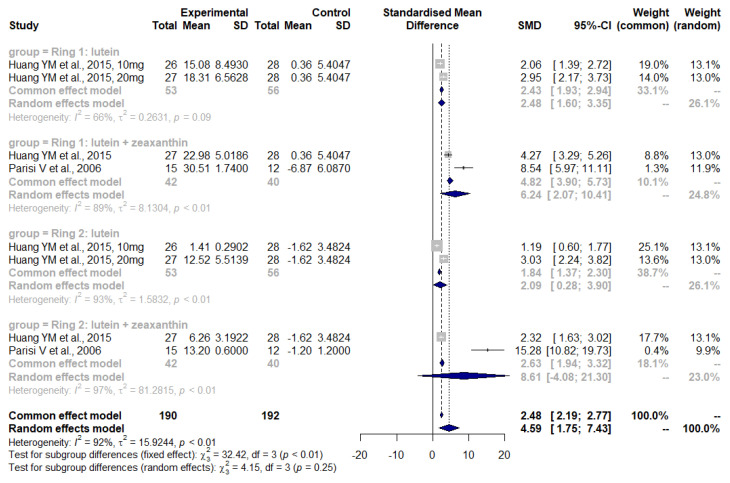
Forest plot of mfERG outcomes in studies where subjects were administered either lutein or lutein plus zeaxanthin; SD, standard deviation; SMD, standardized mean difference; CI, confidence interval [23,24].

Bartlett and Epejersi detected no significant difference in CS between the lutein intervention and the control group (I: −0.02 ± 0.18 log units, C: +0.07 ± 0.07 log units, *p* = 0.388) [36]. Weigert et al. observed a significantly increased MPOD in the lutein intervention group as compared to the control (27.9% ± 2.9%, *p* < 0.01) [31]. A non-significant tendency towards an improvement was seen for VA. Ma et al., Murray et al., and Richer et al. were included in the meta-analysis of the BCVA measurement (Figure 3) [22,27,37]. Huang et al. measured MPOD and mfERG [23]. With respect to MOPD, the first changes, evident after 24 weeks, were seen only in the 20 mg lutein group (baseline: 0.315 ± 0.122 to week 24: 0.395 ± 0.120 optical density units (ODU), *p* < 0.01), while changes in the 10 mg lutein group were only observed after the two years’ visit (baseline: 0.307 ± 0.142 to 2-year follow up: 0.442 ± 0.127 ODU, *p* < 0.001). Changes in MPOD in the zeaxanthin plus lutein group were detected after 48 weeks (baseline: 0.320 ± 0.118 to 48 weeks: 0.384 ± 0.125 ODU, *p* < 0.05). Huang’s mfERG results were combined with those of Parisi et al. in the meta-analysis (Figure 4). Murray et al. noticed a change in MOPD in the lutein group but not in the placebo group (from 0.38 ± 0.19 to 0.53 ± 0.22 ODU, *p* < 0.001). Richer et al. observed an increase in MPOD of about 0.09 log units in the lutein and also in the lutein plus antioxidant groups, in contrast to a small decrease of 0.03 log units in the placebo group (*p* = 0.03, overall differences) after 12 months [22]. Furthermore, there was a significant improvement in the lutein group (*p* = 0.01) after eliminating six subjects from the placebo group found to be consuming a high amount of lutein.

Beatty et al. found a steadily increasing difference in BCVA after 24 months favoring the intervention group by 1.4 letters read (*p* = 0.04); however, this was not considered to be clinically significant [38]. No significance was evident in CS or the AMD progression, although almost every second patient in the placebo group (47.4%) experienced a progression whereas in the intervention group, the value was 41.7%.

Piermarocchi et al. showed a significant difference in BCVA after 24 months (I: 81.4 ± 7.2 scores (logMAR scale), C: 76.8 ± 8.9 scores, *p* = 0.003) [34]. The CS improved in the intervention group as well (*p* = 0.001), and the vision-related quality of life questionnaires improved by 4.6 points (Pelli-Robson chart) (Cl: 2.79, 6.57) after 12 months and by 3.6 points (Cl: 0.5, 6.81) after 24 months whereas there was a worsening in the control group (12 months: −2.5 points, (Cl: −10.12, 5.10), 24 months: −8.7 points (Cl: −16.54, −0.97).

Parravano et al. observed a significant increase in mfERG responses in rings 1 and 2 (*p* < 0.05) but no changes in rings 3–5 [30]. In addition, the SD-OCT results were not significantly different after six months.

Figure 3 shows the effect of carotenoid supplements using the BCVA measurements. The overall effect of carotenoids revealed no significant differences between the intervention and the control groups (SMD: −0.74, 95% CI: −1.87, 0.39). The heterogeneity was high and significant (*I*^2^ = 93%, τ^2^ = 1.5756, *p* < 0.01). The subgroup analysis for lutein also revealed no significant differences between the groups (SMD: −0.86, 95% CI: −2.30, 0.57) and the heterogeneity was high as well (*I*^2^ = 95%, τ^2^ = 2.0596, *p* < 0.01).

Figure 4 was produced by assessing those studies involving carotenoids and an mfERG outcome measurement. This meta-analysis detected an overall effect in the intervention group (combining rings 1 and 2) using mfERG (SMD: 4.59, 95% CI: 1.75, 7.43) with high heterogeneity (*I*^2^ = 92%, τ^2^ = 15.9244, *p* < 0.01). Subgroup analysis has been performed for ring 1 (foveal area) and ring 2 (parafoveal area) with the supplement intervention being lutein or lutein plus zeaxanthin. The subgroup analysis for ring 1 revealed a significant improvement of mfERG in the lutein subgroup (SMD: 2.48, 95% CI: 1.65, 3.35) as well as in the lutein plus zeaxanthin group (SMD: 6.24, 95% CI: 2.07, 10.41). Both analyses had high heterogeneity (*I*^2^ = 66%, τ^2^ = 0.2631, *p* < 0.01) and (*I*^2^ = 89%, τ^2^ = 1.1304, *p* < 0.01) respectively. In ring 2, only the lutein group had a significant effect (SMD: 2.09, 95% CI: 0.28, 3.90) but high heterogeneity (*I*^2^ = 93%, τ^2^ = 1.5832, *p* < 0.01)

### 3.5. Lutein and Zeaxanthin Plus n-3 LC-PUFA

For this supplement combination, three of the four studies measuring BCVA could be included in the meta-analysis [26,29,39]. Berrow et al. detected no significant change in VA and CS between the intervention and control group after follow-up [28]. In addition, no clinical significance was observed in mfERG outcomes. Dawczynski et al. also measured MPOD as well as BCVA, [26]. In both intervention arms (I1: 10 mg lutein, I2: 20 mg lutein), the MPOD volume increased significantly (*p* < 0.001), whereas in the control group it declined significantly (*p* = 0.04). Changes in all MPOD parameters were significantly different between I1 and control as well as between I2 and control (*p* < 0.001). García-Layana et al. also observed significant differences between the intervention and control in MPOD (I: +0.162 units, C: +0.059 units, *p* < 0.05) [29]. Piatti et al. analyzed the AMD progression in the intervention and control groups and found a significant difference between both groups (I: 2.1% progression, C: 15.4% progression, *p* < 0.05) [39].

**Figure 5:**
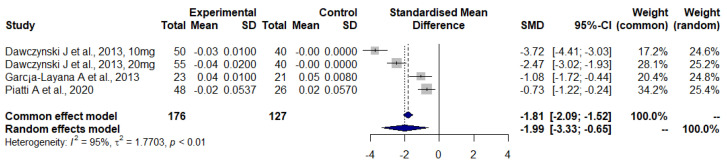
Forest plot of BCVA in studies with lutein and zeaxanthin combined with *n*-3 LC-PUFA; SD, standard deviation; SMD, standardized mean difference; CI, confidence interval *n-3* LC-PUFA (DHA and EPA) [26,29,39].

Figure 5 shows the effect of lutein plus zeaxanthin supplements, including n3 LC-PUFA using the BCVA values (logMar scale). This meta-analysis points to a significant improvement in BCVA in the intervention group as compared to the control group (SMD: −1.99, 95% CI: −3.33, −0.65). The heterogeneity here was high (*I*^2^ = 95%, τ^2^ = 1.7703, *p* < 0.001).

In their pilot study, Querques et al. 2009, detected no statistical difference for the mean BCVA value in patients with mild AMD in one eye, and more severe disease in the other eye, when followed with or without supplementation for six months [35]. No progression in either central geographic atrophy or MNV was observed. OCT examinations revealed no statistically significant differences in drusenoid pigment epithelium detachment between the intervention and control group in the six months of the trial (I: 139.17 to 148.42 µm vs. C: 140.34 to 184.29 μm) (*p* > 0.05).

Drusen remodeling had not changed significantly between the *n*-3 LC-PUFA as compared to the control group after three years in the NAT2 study of Querques et al. published in 2016 [32]. However, total drusen diameter reduction in the inner subfield was significantly associated with age (older patients: r = −0.17; *p* = 0.003). In addition, female gender was associated with a decreased total drusen diameter in the inner subfield (−1.07 ± 123.8, *p* = 0.03).

Examining the same study subjects as in Querques et al. 2016, Souied et al. detected no significant differences in time to occurrence and incidence of MNV between the *n*-3 LC-PUFA group (19.5 ± 10.9 months and 28.4%, respectively) and the placebo group (18.7 ± 10.6 months and 25.6%), respectively [40].

### 3.6. Zinc

In the study conducted by Newsome et al., BCVA improved significantly after 6 months in the intervention group (right eye: +4.405 ± 0.712, left eye: +3.057 ± 0.639, number of letters) as compared to the control group (right eye: −1.054 ± 0.489, left eye: −0.703 ± 0.642) (*p* < 0.001) [41]. In addition, the CS using the Pelli-Robson contrast sensitivity chart improved in the intervention (right eye: +0.199 ± 0.045, left eye: −0.039 ± 0.029) but worsened in the control group (right eye: 0.159 ± 0.029, left eye: −0.035 ± 0.025) group (*p* < 0.001).

The AREDS research group observed a statistically significant reduction in the odds ratio (OR) for AMD categories 2, 3 and 4 to progress to advanced AMD development in all three intervention groups in comparison to the control group [13]. The OR for antioxidant plus zinc were as follows: 0.72 (99% Cl, 0.52–0.98), zinc alone: 0.75 (99% CI, 0.55–1.03) and antioxidants alone: 0.80 (99% CI, 0.59–1.09). Patients from category 2 had only a 1.3% 5-year probability of progression to advanced AMD. After excluding this category, the OR estimates increased (antioxidants plus zinc: OR, 0.66; 99% CI, 0.47–0.91; zinc: OR, 0.71; 99% CI, 0.52–0.99; antioxidants: OR, 0.76; 99% CI, 0.55–1.05). Nonetheless, only in the subjects receiving antioxidants plus zinc was there a statistically significant reduction in the rates of at least moderate VA (OR, 0.73; 99% CI, 0.54–0.99).

### 3.7. Curcumin

Allegrini et al. reported a significantly improved BCVA (*p* < 0.05) after curcumin treatment in wet AMD (0.40 logMAR [0.28–0.4]) as compared to the control group (1.0 logMAR [0.46–1.5]) but no significant change in central macular thickness [33]. In addition, the numbers of anti-vascular endothelial growth factor (VEFG) injections were significantly reduced during the intervention (intervention: 4 injections, control 7 injections, on average during the intervention).

## 4. Discussion

Our meta-analysis and SR aimed to analyze if certain dietary supplements could delay the progression of AMD or even exert an improvement in any AMD stages by examining different parameters measuring visual and retinal functions. However, due to the diverse outcome measurements for visual functions and the various supplements used in the trials, only a few studies could be included into the meta-analyses. Therefore, three meta-analyses were conducted with carotenoid supplementations, i.e., lutein and zeaxanthin (with and without *n*-3 LC-PUFA) which had reported measurements of BCVA and mfERG.

According to the results of our meta-analysis with respect to the BCVA measurement (Figure 3), the overall effect of the carotenoids was not significant. The use of lutein alone did not significantly affect the BCVA values of the AMD patients. No conclusion about the combination of lutein plus zeaxanthin could be drawn due to a lack of studies. However, some effects on BCVA were found for the supplementation with lutein plus zeaxanthin in two studies [34,38] and these were accompanied with improvements in both CS and quality of life [34]. These interventions lasted for two years, which may imply that a longer supplementation period improves visual function.

Furthermore, supplementation with lutein and lutein plus zeaxanthin improved retinal function when assessed with the mfERG method (Figure 4) [23,24]. The meta-analysis of the effect of lutein on retinal function using mfERG revealed some interesting details. An overall effect was observed, and subgroup analyses showed that both lutein on its own and lutein plus zeaxanthin supplementation effectively enhanced ring 1 and ring 2 results. However, enhanced mfERG results do not necessarily correlate directly with visual acuity and the quality of life [42]. As the mfERG mainly measures the function of the foveal cone and bipolar cells in the retina, it could be postulated that the carotenoids can positively affect the functions of cone and bipolar cells.

The meta-analysis also showed that the administration of lutein and zeaxanthin did not exert the same effect as lutein alone in the mfERG results when measuring ring 2 (parafoveal) function [23,24]. It has been shown that simultaneous administration of different types of carotenoids might prevent the absorption of these supplements from the intestine and decrease the effectiveness of these compounds (reviewed by Berg H et al. and Castenmiller J et al. [43,44]. For example, this has been shown for β-carotene and lutein. Thus, in the future, based on these pharmacokinetic considerations, it could be justified to study the effectiveness of lutein and zeaxanthin separately.

The meta-analysis considering simultaneous administration of carotenoids (lutein, astaxanthin, and zeaxanthin) and *n*-3 LC-PUFA revealed a significant improvement in BCVA (Figure 5). In addition, Dawczynski et al. and García-Layana et al. demonstrated an increase in the MPOD values reflecting the accumulation of carotenoids into macular pigment [26,29]. These results imply that the simultaneous use of *n*-3 LC-PUFA and carotenoids might be beneficial in preventing the progression of AMD and the resulting deterioration of visual function. Moreover, it has been shown that the co-consumed fatty acids can alter the absorption of xanthophyll, with the effect varying on the lipid source (reviewed by Moran N et al. [45]). Therefore, the phenomenon mentioned above that fatty acids can alter the absorption of xanthophyll might explain the variable results when comparing the effectiveness of carotenoid formulations with or without *n*-3 LC-PUFA supplementation. However, additional vitamin and mineral compounds, which were present in almost all carotenoid formulations except for those of Huang et al., Ma et al., and Weigert et al., might also affect the absorption and metabolism of other compounds in the supplements, and these interactions could explain the variations in the progression of AMD and the assessed visual parameters [23,27,31].

The effect of carotenoid supplementation on other parameters measuring retinal function and appearance showed an improvement in MPOD depending on the dose and duration of the supplementation [22,23,31,37]. Typically, higher doses of lutein supplementation (20 vs. 10 mg/day) were associated with increased MPOD values. In addition, Ma et al. reported that the MPOD increases after supplementation are especially emphasized in patients with low macular pigmentation at baseline [27]. Furthermore, an increased risk of AMD progression among patients with low MPOD has been observed [46]. Bone et al. detected this phenomenon by measuring the amounts of lutein and zeaxanthin from donor eyes suffering from AMD [46]. In addition, Murray et al., Ma et al., Richer et al., and Weigert et al. discussed if the improvement in the visual function in their studies could have been attributed to the increased MPOD [22,27,31,37]. Thus, administering carotenoids during the early stages of AMD might have a preventive effect on the progression of AMD, and in addition, measuring the change in MPOD could possibly produce important information about the risk of AMD.

Querques et al. and Souied et al. did not detect any statistical significance in any visual parameters and drusen remodeling when AMD patients were supplemented with *n*-3 LC-PUFA. It should be noted that vitamin E was present in these two studies without any significant effect on outcome measurements [32,40]. However, the patient populations in these trials consisted of patients already having advanced wet AMD [32,35,40]. Thus, it can be postulated that *n*-3 LC-PUFA supplementation would benefit patients in the earlier stages of AMD.

Both studies using zinc supplements have observed positive effects in BCVA or AMD progression [13,41]. Furthermore, another SR with a meta-analysis investigating zinc supplements and dietary zinc intake concluded that this metal exerted a possible positive effect on AMD progression in all stages of AMD [47]. However, it was zinc in combination with antioxidants rather than zinc alone that achieved significant results in visual acuity. The AREDS study also observed the slowest AMD progression in the combination of zinc and antioxidants compared to the administration of zinc or antioxidants on their own [13]. The exact mechanism by which zinc protects against AMD is somewhat elusive. However, Blasiak et al. speculated that the protective effects of zinc might be attributed to a modulation of the deficient autophagy present in AMD [48].

Curcumin, another oxidant compound which has been combined with resveratrol, was administered in one of the selected studies and was associated with improvements in BCVA and a decrease in the number of anti-VFGF injections [33]. Laboratory studies have indicated that the positive impact of curcumin on AMD is mediated through a decrease in the apoptotic rates of retinal pigment epithelial (RPE) cells, VEGF inhibition, and decreasing the overall extent of inflammation [49]. Since curcumin is very poorly absorbed from the gastrointestinal tract (90% is excreted into feces), any effects in peripheral tissue might be derived from its metabolites [50]. Furthermore, the resveratrol present in this formulation also possesses potentially beneficial effects in treating AMD due to its high antioxidant capacity (reviewed extensively by Salehi B et al. [51]). In pre-clinical studies, resveratrol has been demonstrated to downregulate VEGF (as reviewed by Gliemann L et al. [52]), which is the current pharmacological treatment strategy for wet AMD [11]. However, no definitive conclusion about the efficacy of curcumin alone can be drawn, but since the results from Allegrini et al. are promising, further clinical studies are warranted. One clinical study focusing on curcumin’s effects on drusen in AMD patients was completed in November 2021 (clinicalTrials.gov: NCT04590196). Its results, in conjunction with other future well-performed clinical studies, might give a more detailed insight into the effect of curcumin.

The heterogeneity in all meta-analyses was high. On the one hand, this can be explained by the low number of studies; on the other hand, several factors such as ethnicity, BMI, or education status can bias the study outcomes. For example, our meta-analyses included studies from different ethnic groups, which may have influenced the overall result. Several investigators have demonstrated that the prevalence of early and late AMD varies in different racial/ethnic groups. Thus, one study claimed that the highest AMD prevalence occurs in the Caucasian and Chinese populations [53]. Other findings in multiethnic Asian populations have suggested that visual specific function is subject to an independent ethnic influence [54]. In addition, carotenoid-specific metabolic enzymes and ocular carotenoid-binding proteins may modulate the proportion of carotenoids reaching the retina and macula [27]. Hence, between-race variations in these proteins may affect the outcome. In addition, it is well known that AMD has a genetic component which means that in the future it may be possible to develop individualized treatment options [55,56]. Another factor which could be considered, BMI, might influence AMD prevalence/progression although the results are contentious. A higher incidence of late AMD but not early AMD was observed among obese subjects [57]. Another study found no association of body weight alone but there was an association with weight and smoking in an Indian population [58]. A recent systematic review showed evidence of a positive association between BMI-defined obesity and AMD in Western populations but not in Asian populations [59]. Additionally, physical activity has been claimed to display a protective association with the incidence and progression of AMD [60].

In addition to the administration of cholesterol-lowering medication i.e., statins, which seem to be protective for early and wet AMD [59,60], it has been thought that the individual’s social-economic status may influence his/her risk of developing AMD [61]. People with a low income have a higher prevalence of AMD, partly explained by the lower nutritional quality of their diet [61,62,63]. Interestingly, whereas one study found a positive correlation with low education [62], a recent study showed an association between AMD prevalence and high academic qualifications [61].

Taking everything together, it does seem that the incidence and progression of AMD are influenced by many factors which could modify the outcomes of nutrient supplementation studies and should be taken into account in upcoming trials, since most of the included studies did not consider these confounding factors in their analyses.

### 4.1. Clinical Relevance

According to common clinical principles, the diagnosis of AMD is currently based on BCVA, fundus images, OCT, and fluorescein angiography [64]. However, the studies included in this SR involved many and diverse outcome measurements. Nevertheless, only studies with BCVA and mfERG as outcome measurements were included in the meta-analysis since the scale and units of these techniques are uniform. Measuring BCVA is relatively straightforward in basic clinical settings and does not require specific instruments in the assessment as compared to mfERG, where elaborate instruments and well-trained personnel are required. However, mfERG can detect early changes in the macular function related to AMD. Both of these methods have advantages and disadvantages but their inabilities to measure precisely the extent of the damage caused by AMD limits their use as research methods. Thus, it would be important to harmonize the scope of methods used when studying the effect of nutritional supplements on AMD progression. The changes in the clinical state of subjects are most likely small, warranting precise interpretation throughout long intervention periods. It would also be important that the used outcome measurement methods should be available for most researchers.

There are currently no available treatment options for dry AMD. Furthermore, the current treatment for wet AMD with intravitreal injections of anti-VEGF agents is ineffective for some patients, and serious side effects are possible [65]. In addition, this anti-VEGF treatment is expensive as the cost of the cheapest anti-VEGF agent, bevacizumab, is 50 €/injection, but it rises to hundreds of €/injection for the other drugs in use [66]. Millions of anti-VEGF injections are given intravitreally monthly or bi-monthly worldwide by health care professionals for the treatment of posterior eye diseases [12]. It is evident that there is a huge need for more efficient and cost-effective treatment alternatives. Nutritional supplements are relatively cheap and safe within the recommended dose range with good patient compliance. There is also evidence from large RCTs supporting the use of nutritional supplements in the treatment of AMD. The AREDS study demonstrated that patients consuming high amounts of antioxidants together with zinc and copper were less likely to develop advanced AMD [13]. The following AREDS2 further showed that patients with intermediate AMD did benefit incrementally when a combination of lutein and zeaxanthin was administered with the antioxidants [14]. However, adding *n*-3 LC-PUFA or lutein and zeaxanthin into the original AREDS formulation which contained beta-carotene did not have any additional benefit on the overall risk for the progression of AMD. These commonly accepted findings support the results of our meta-analysis. However, the benefit of *n*-3 LC-PUFA supplementation when combined with lutein and zeaxanthin was revealed in our meta-analysis of patients with early, dry and intermediate AMD (Figure 5). This is interesting as the AREDS study did not detect any evidence for this kind of effect when the AREDS formulation was administered for patients with early AMD [13]. In addition, AREDS2′s results did not find any additional benefit for enhancing the original AREDS formulation with *n*-3 LC-PUFA or lutein + zeaxanthin. However, the population in AREDS2 was restricted to patients with intermediate AMD, and its complex study design might have masked some potential effects of individual nutrients [14]. Nevertheless, according to very recent data, results from long-term epidemiologic follow-up study up to 10 years of the AREDS2 cohort suggest beneficial effects when replacing beta-carotene with lutein and zeaxanthin [67]. In contrast to beta-carotene, lutein and zeaxanthin had a potential beneficial association with late AMD progression. Beta-carotene usage is known to increase the risk for lung cancer [68], almost doubling the risk according to findings of the AREDS2 study, but there was no statistically significant increased risk linked with lutein and zeaxanthin [67]. These findings further support the positive effects of lutein and zeaxanthin in AMD management and encourage a preference for these xanthophylls over beta-carotene; a factor that should be taken into account when designing future studies with comparable combinations of *n*-3 LC-PUFA, lutein, and zeaxanthin in trial subjects consisting of early AMD patients. As there are currently no medical treatment options for early and dry AMD, high-quality additional information on improving or halting the AMD disease progression by nutritional supplements would represent a breakthrough for finding a cost-effective therapy capable of preventing the progression of AMD to its advanced stages. Even slowing down the disease progression from advancing to wet AMD by a few years would save costs remarkably.

### 4.2. Strength and Limitations

The strength of this study is its systematic approach and carefully subdivided supplement categories and subgroup analyses which enhance the study’s ability to reveal the effects of the individual supplements. The major limitation is the rather low number of studies included in the meta-analysis. Due to this, the heterogeneity is very high, and the interpretation of the results has to be carried out with caution. The limitations of the meta-analysis and SR also include the diverse nature of the compositions of the nutritional supplement provided in these studies, as the presence of several different vitamins and minerals limits the interpretation of the effect of each individual nutritional compound. Even though a careful consideration in pooling the studies for meta-analysis was used, the possibility of confounding factors such as demographical data and intervention design cannot be fully ruled out. Differences in ethnicities and BMI values of patients included in the meta-analysis contribute to the heterogeneity but also reflect rather well the AMD patient population as a whole. Thus, in the future, it is recommended to design studies with a well-defined patient population, a predetermined duration of the intervention and adopting the use of promising supplement combinations. One example would be to treat patients with early AMD with a combination of *n*-3 LC-PUFA, lutein, and zeaxanthin.

## 5. Conclusions

Combinations of carotenoids, namely lutein, and zeaxanthin, with *n*-3 LC-PUFA might have the best effect on improving BCVA of patients with early or intermediate AMD. These carotenoids alone seem to have no effect, at least if administered for shorter periods (less than one year). Longer interventions are more likely to show positive effects, but further studies are warranted to clarify whether long-term supplementations with carotenoids can modify the course of AMD. Due to a lack of studies with other supplements, no final conclusion can be drawn. Nevertheless, since they represent the whole AMD patient population, the results from this meta-analysis can be used as a basis for future clinical trials e.g., administering promising nutritional supplement combinations to well-specified patient populations.

Several of the retrieved studies provided their test subjects with formulations containing many different compounds. This makes it difficult to conclude if any of the effects were attributable to a single compound, whereas if the compounds exerted a synergistic effect, this might lead to improvements in the course of AMD. Finally, we propose that in the future, there should be a consensus reached on appropriate outcome measurements in AMD so that the outcomes of different trials will be truly comparable.

## Figures and Tables

**Table 1:** Publications selected for the systematic review, including study characteristics.

First Author, Year of Publication, Country	Study Design	Sample Size (F/M)	Mean Age (SD) (Years)	AMD Stage	Dietary Supplements (Total Daily Dose)	Control	Outcome Measurements	Intervention Duration (Months)
Bartlett HE and Epejersi F 2007, UK [36]	double-masked RCT	I: 13 (7/6) C: 7 (4/3)	I: 69.2 (7.8) C: 69.2 (7.8)	ARM, atrophic AMD	6 mg L, retinol, vitamin C, vitamin E, Zn, Cu	placebo (cellulose)	CS (Pelli-Robson chart)	9
Weigert G et al. 2011, Austria [31]	RCT	116 (66/50)	71.6 (8.6)	AREDS stages 2, 3 and 4	month 1–3: 20 mg L month 4–6: 10 mg L	placebo	MPOD, VA, MDLT	6
* Ma L et al. 2012, China [27]	RCT	I1: 26 (16/10) I2: 27 (15/12) I3: 27 (15/12) C: 27 (16/11)	I1: 69.9 (8.4) I2: 69.0 (6.8) I3: 68.6 (7.0) C: 68.9 (7.6)	early AMD	I1: 10 mg L I2: 20 mg L I3: 10 mg L + 10 mg Z	placebo	MPOD, BCVA, CS, photo recovery time, Amsler grid	10.5
* Murray IJ et al. 2013, UK [37]	double-masked RCT	I: 36 (20/16) C: 37 (24/12)	I: 71.9 (8.7) C: 69.2 (8.6)	early AMD	10 mg L	placebo soya bean oil capsula	MPOD, BCVA	12
* Richer SP et al. 2004, USA [22]	RCT	I1: 29(2/27) I2: 30(1/29) C: 31(1/30)	I1: 74.4 (6.4) I2: 73.5 (8.5) C: 76.1 (6.4)	atrophic AMD	I1: 10 mg L (FloraGlo) I2: 10 mg L, antioxidants, vitamins, minerals (OcuPower)	placebo (maltodextrin)	MPOD, near + distance VA, glare recovery, CS, AMD retinopathy differences	12
* Huang YM et al. 2015, China [23]	double-masked RCT	I1: 26 (17/9) I2: 27 (14/13) I3: 27 (15/12) C: 28 (17/11)	I1: 69.7 (8.3) I2: 69.3 (6.9) I3: 68.5 (6.9) C: 69 (7.5)	early AMD	I1: 10 mg L I2: 20 mg L I3: 10 mg L + 10 mg Z	placebo	MPOD, mfERG, Microperimetry	24
* Parisi V et al. 2008, Italy [24]	RCT	I: 15 (9/6) C1: 12 (6/6) C2: 15 (9/6)	I: 69.4 (4.3) C1: 69.7 (6.2) C2: 69.6 (5.1)	AREDS stage 3	10 mg L, 1 mg Z, 4 mg AX, vitamin C, vitamin E, Zn, Cu	C1: no supplements C2: healthy age-matched subjects	mfERG	12
Beatty S et al. 2012, Ireland [38]	double-masked RCT	I: 216 (124/92) C: 217 (124/93)	≥55 (NA)	early AMD	12 mg L, 0.6 mg Z, vitamin E, vitamin C, Zn, Cu gluconate	placebo	BCVA, CS (Pelli-Robson chart), AMD grade using fundus photographs, Raman spectroscopy counts	12–36
Piermarocchi S et al. 2011, Italy [34]	open-labeled RCT	I: 103 (62/41) C: 42 (25/17)	I: 72.5 (6.8) C: 72.6 (7.5)	dry AMD	10 mg L, 1 mg Z, 4 mg AX, vitamin C, vitamin E, ZN, Cu,	no supplement	BCVA, CS (Pelli-Robson chart), visual function via the Italian-validated version of the 25-item NEI VFQ test	24
Parravano M et al. 2019, Italy [30]	double-masked RCT	I: 15 (NA) C: 15 (NA)	I: 68.5 (8.8) C: 70.1 (9.9)	AREDS stage 3	20 mg L, 4 mg Z, N-acetylcysteine, vitamins, minerals, rutin (2 tablets Marcuprev/day)	placebo with cellulose	mfERG and SD-OCT	6
Berrow EJ et al. 2013, UK [28]	blinded RCT	I: 8 (NA) C: 6 (NA)	I: 70 (7.5) C: 65.5 (9.3)	ARM	12 mg L, 0.6 mg Z, EPA 240 mg, DHA 840 mg, Vitamin C, Cu oxide, Vitamin E, Zn (Ocuvite Duo)	placebo	mfERG, VA, CS	13
* Dawczynski J et al. 2013, Germany [26]	double-masked RCT	I1: 50 I2:55 C:40 overall: (79/66)	69 (10)	dry AMD	I1: 10 mg L + 1 mg Z, antioxidants, DHA (1 tablet FloraGLO^®^/day) I2: 2 tablets FloraGLO^®^/day	placebo	MPOD, BCVA	12
* García-Layana A et al. 2013, Spain [29]	RCT	I: 23 (12/10) C: 21 (13/8)	I: 69.2 (7.8 SEM) C: 67.8 (9.2 SEM)	early AMD	12 mg L, 0.6 mg Z, 280 mg of DHA	placebo (sugar)	MPOD, BCVA, CS, OCT	12
* Piatti A et al. 2020, Italy [39]	double-masked RCT	I: 48 (31/17) C: 26 (20/6)	I: 71.4 (6.5) C: 72.7 (5.5)	intermediate AMD	10 mg L, 4 mg AX, 2 mg Z, vitamin C, vitamin E, Zn, Cu, fish oil 500 mg (EPA 185 mg + DHA 140 mg)	placebo	retinography, BCVA	24
Querques G et al. 2009, France [35]	Comparative pilot study	38 (28/10)	72.74 (6.25)	wet AMD	720 mg EPA and 480 DHA mg (fish oil capsule)	no supplement	BCVA, FA, OCT	6
Querques G et al. 2016, France [32]	RCT	I: 87 (59/28) C: 80 (44/36)	I: 74.4 (6.7) C: 72.8 (6.9)	wet AMD in one eye, ARM in the study eye	840 mg DHA, 270 mg EPA, 6 mg vitamin E	placebo (602 mg olive oil)	drusen burden and disease progression by fundus photography	36
Souied E et al. 2013, France [40]	RCT	I: 134 (92/42) C: 129 (78/51)	I: 73.9 (6.6) C: 73.2 (6.8)	wet AMD	840 mg DHA, 270 mg EPA, 6 mg vitamin E per day (3 Reti-Nat1-capsules/day)	placebo (602 mg olive oil)	CNV progression + drusen formation by fundus photography, BCVA	36
Newsome D et al. 2008, USA [41]	RCT	I: 37 (30/7) C: 37 (29/8)	I: 72.1 (11.7) C: 73.3 (9.5)	dry AMD	50 mg Zn (as monocysteine)	placebo (cellulose)	BCVA, CS, photo recovery time	6
AREDS research group et al. 2001, USA [13]	RCT	I1: 936 (55/881) I2: 897 (57/840) I3: 882 (56/826) C: 894 (56/838)	I1: 69 (NA) I2: 70 (NA) I3: 69 (NA) C: 69 (NA)	all 4 AREDS stages	I1: antioxidants 500 mg Vitamin C, 400 IU vitamin E, 15 mg Beta carotene I2: 80 mg Zn, 2 mg Cu oxide I3: antioxidants + Zn	placebo	fundus photographs	follow-up for 6.3 years
Allegrini D et al. 2021, Italy [33]	Controlled retrospective study	I: 18 (6/12) C: 24 (10/14)	I: 80 (75–87 IQR) C: 80 (78–86 IQR)	wet AMD	50 mg of curcumin, AREDS2 components, 4 mg AX, 20 mg resveratrol	no supplement, intravitreal injections of anti-VEGF (aflibercept)	BCVA, CMT	6

AMD, age-related macular degeneration; AREDS, age-related eye disease study; ARM, age-related maculopathy; AX, astaxanthin; BCVA, best-corrected visual acuity; C, control group; CMT, central macular thickness; CS, contrast sensitivity; Cu, copper, DHA, docosahexaenoic acid; EPA, eicosapentaenoic acid; ERG, electroretinogram; F, female; FA, fluorescence angiography; FAF, fundus autofluorescence; I, intervention group; IQR, interquartile range; L, lutein; M, male; MDLT, mean differential light threshold; mfERG, multifocal electroretinogram; MPOD, macular pigment optical density; NA, not available; OCT, optical coherence tomography; RBC, red blood cell membrane; RCT, randomized controlled trial; SD, standard deviation; SD-OCT, spectral domain optical coherence tomography; SEM, standard error of mean; VA, visual acuity; Z, zeaxanthin; Zn, zinc, * studies included into the metanalysis.

## Data Availability

Access to the data, R codes, and/or material can be sought via contacting the responsible authors.

## Supplementary Materials

The following supporting information can be downloaded at: https://www.mdpi.com/article/10.3390/nu14204273/s1, Supplementary File S1: Search terms, Supplementary File S2: Table of listed supplement combinations.
