# Supplementary material for: The Effect of Dietary Supplementations on Delaying the Progression of Age-Related Macular Degeneration: A Systematic Review and Meta-Analysis
Source: Nutrients. 2022 Oct 13;14(20):4273. doi: 10.3390/nu14204273 (PMC9610847; doi:10.3390/nu14204273)
Supplement: Supplementary file 1 [file nutrients-14-04273-s001.zip › nutrients-1940203-supplementary.pdf]

Supplementary Information (SI):

SI1: Search string and number of results

Search date: October 8, 2021
PubMed
#1 "Macular Degeneration"[mh] OR "Geographic Atrophy"[mh]
26,518 results
#2 "macular degeneration"[tw] OR "retinal degeneration"[tw] OR "macular dystroph*"[tw] OR maculopath*[tw] OR "geographic atrophy"[tw] OR "dry amd"[tw] OR "wet amd"[tw] OR "exudative amd"[tw]
44,614 results
#3 #1 OR #2
51,745 results
#4 "beta Carotene"[mh] OR Vitamins[mh] OR Antioxidants[mh] OR Nutrients[mh] OR "Dietary Supplements"[mh] OR Zinc[mh] OR Lutein[mh] OR Zeaxanthins[mh] OR "Fatty Acids"[mh]
795,915 results
#5 "beta carotene"[tw] OR betacarotene[tw] OR vitamin*[tw] OR antioxidant*[tw] OR nutrient*[tw] OR micronutrient*[tw] OR "dietary supplement*"[tw] OR "diet supplement*"[tw] OR "nutrition supplement*"[tw] OR "nutritional supplement*"[tw] OR zinc[tw] OR zn[tw] OR lutein[tw] OR zeaxanthin*[tw] OR "fatty acid*"[tw] OR "eicosapentaenoic acid*"[tw] OR epa[tw] OR "docosahexaenoic acid*"[tw] OR dha[tw] OR "unsaturated fat*"[tw] OR "polyunsaturated fat*"[tw] OR "n 3 pufa*"[tw] OR "n3 pufa*"[tw] OR "n 3 fat*"[tw] OR "n3 fat*"[tw] OR "n 3 oil*"[tw] OR "n3 oil*"[tw] OR "omega 3"[tw] OR omega3[tw] OR "n 6 pufa*"[tw] OR "n6 pufa*"[tw] OR "n 6 fat*"[tw] OR "n6 fat*"[tw] OR "n 6 oil*"[tw] OR "n6 oil*"[tw] OR "omega 6"[tw] OR omega6[tw]
1,186,432 results
#6 #4 OR #5
1,493,612 results
#7 "Randomized Controlled Trials as Topic"[mh] OR "Random Allocation"[mh] OR "Randomized Controlled Trial"[pt] OR "Clinical Trials as Topic"[mh] OR "Clinical Trial"[pt] OR "Double-Blind Method"[mh] OR Placebos[mh] OR "Cohort Studies"[mh]
3,254,832 results
#8 rct[tw] OR rcts[tw] OR "randomized controlled"[tw] OR "randomised controlled"[tw] OR "random control"[tw] OR "randomized trial*"[tw] OR "randomised trial*"[tw] OR "random allocation"[tw] OR randomly[tw] OR "clinical trial*"[tw] OR "controlled trial*"[tw] OR "control trial*"[tw] OR "double blind*"[tw] OR "double mask*"[tw] OR placebo*[tw] OR "cohort stud*"[tw] OR "cohort analysis"[tw] OR "follow up stud*"[tw] OR "longitudinal stud*"[tw] OR "prospective stud*"[tw] OR "retrospective stud*"[tw]
4,014,897 results
#9 #7 OR #8
4,018,026 results
#10 #3 AND #6 AND #9
800 results (final results)
Scopus

#1 TITLE-ABS-KEY("macular degeneration" OR "retinal degeneration" OR "macular dystroph*" OR maculopath* OR "geographic atrophy" OR "dry amd" OR "wet amd" OR "exudative amd")
55,860 results
#2 TITLE-ABS-KEY("beta carotene" OR betacarotene OR vitamin* OR antioxidant* OR nutrient* OR micronutrient* OR "dietary supplement*" OR "diet supplement*" OR "nutrition supplement*" OR "nutritional supplement*" OR zinc OR zn OR lutein OR zeaxanthin* OR "fatty acid*" OR "eicosapentaenoic acid*" OR epa OR "docosahexaenoic acid*" OR dha OR "unsaturated fat*" OR "polyunsaturated fat*" OR "n 3 pufa*" OR "n3 pufa*" OR "n 3 fat*" OR "n3 fat*" OR "n 3 oil*" OR "n3 oil*" OR "omega 3" OR omega3 OR "n 6 pufa*" OR "n6 pufa*" OR "n 6 fat*" OR "n6 fat*" OR "n 6 oil*" OR "n6 oil*" OR "omega 6" OR omega6)
2,524,001 results
#3 TITLE-ABS-KEY(rct OR rcts OR "randomized controlled" OR "randomised controlled" OR "random control" OR "randomized trial*" OR "randomised trial*" OR "random allocation" OR randomly OR "clinical trial*" OR "controlled trial*" OR "control trial*" OR "double blind*" OR "double mask*" OR placebo* OR "cohort stud*" OR "cohort analysis" OR "follow up stud*" OR "longitudinal stud*" OR "prospective stud*" OR "retrospective stud*")
4,960,703 results

#4 #1 AND #2 AND #3
1200 results (final results)

Web of Science

#1 TS=("macular degeneration" OR "retinal degeneration" OR "macular dystroph*" OR maculopath* OR "geographic atrophy" OR "dry amd" OR "wet amd" OR "exudative amd")
47,598 results
#2 TS=("beta carotene" OR betacarotene OR vitamin* OR antioxidant* OR nutrient* OR micronutrient* OR "dietary supplement*" OR "diet supplement*" OR "nutrition supplement*" OR "nutritional supplement*" OR zinc OR zn OR lutein OR zeaxanthin* OR "fatty acid*" OR "eicosapentaenoic acid*" OR epa OR "docosahexaenoic acid*" OR dha OR "unsaturated fat*" OR "polyunsaturated fat*" OR "n 3 pufa*" OR "n3 pufa*" OR "n 3 fat*" OR "n3 fat*" OR "n 3 oil*" OR "n3 oil*" OR "omega 3" OR omega3 OR "n 6 pufa*" OR "n6 pufa*" OR "n 6 fat*" OR "n6 fat*" OR "n 6 oil*" OR "n6 oil*" OR "omega 6" OR omega6)
1,835,613 results
#3 TS=(rct OR rcts OR "randomized controlled" OR "randomised controlled" OR "random control" OR "randomized trial*" OR "randomised trial*" OR "random allocation" OR randomly OR "clinical trial*" OR "controlled trial*" OR "control trial*" OR "double blind*" OR "double mask*" OR placebo* OR "cohort stud*" OR "cohort analysis" OR "follow up stud*" OR "longitudinal stud*" OR "prospective stud*" OR "retrospective stud*")
2,227,088 results
#4 #1 AND #2 AND #3
616 results (final results)

CINAHL (EBSCO)

S1 "macular degeneration" OR "retinal degeneration" OR "macular dystroph*" OR maculopath* OR "geographic atrophy" OR "dry amd" OR "wet amd" OR "exudative amd"
9,432 results
S2 "beta carotene" OR betacarotene OR vitamin* OR antioxidant* OR nutrient* OR micronutrient* OR "dietary supplement*" OR "diet supplement*" OR "nutrition supplement*" OR "nutritional supplement*" OR zinc OR zn OR lutein OR zeaxanthin* OR "fatty acid*" OR "eicosapentaenoic acid*" OR epa OR "docosahexaenoic acid*" OR dha OR "unsaturated fat*" OR "polyunsaturated fat*" OR "n 3 pufa*" OR "n3 pufa*" OR "n 3 fat*" OR "n3 fat*" OR "n 3 oil*" OR "n3 oil*" OR

"omega 3" OR omega3 OR "n 6 pufa*" OR "n6 pufa*" OR "n 6 fat*" OR "n6 fat*" OR "n 6 oil*" OR "n6 oil*" OR "omega 6" OR omega6
191,786 results
S3 rct OR rcts OR "randomized controlled" OR "randomised controlled" OR "random control" OR "randomized trial*" OR "randomised trial*" OR "random allocation" OR randomly OR "clinical trial*" OR "controlled trial*" OR "control trial*" OR "double blind*" OR "double mask*" OR placebo* OR "cohort stud*" OR "cohort analysis" OR "follow up stud*" OR "longitudinal stud*" OR "prospective stud*" OR "retrospective stud*"
1,025,126 results
S4 S1 AND S2 AND S3
183 results (final results)
Cochrane Central Register of Controlled Trials
Issue 10 of 12, October 2021
Cochrane Database of Systematic Reviews
Issue 10 of 12, October 2021
#1 ("macular degeneration" OR "retinal degeneration" OR "macular dystroph*" OR maculopath* OR "geographic atrophy" OR "dry amd" OR "wet amd" OR "exudative amd"):ti,ab,kw
3,836 results
#2 ("beta carotene" OR betacarotene OR vitamin* OR antioxidant* OR nutrient* OR micronutrient* OR "dietary supplement*" OR "diet supplement*" OR "nutrition supplement*" OR "nutritional supplement*" OR zinc OR zn OR lutein OR zeaxanthin* OR "fatty acid*" OR "eicosapentaenoic acid*" OR epa OR "docosahexaenoic acid*" OR dha OR "unsaturated fat*" OR "polyunsaturated fat*" OR "n 3 pufa*" OR "n3 pufa*" OR "n 3 fat*" OR "n3 fat*" OR "n 3 oil*" OR "n3 oil*" OR "omega 3" OR omega3 OR "n 6 pufa*" OR "n6 pufa*" OR "n 6 fat*" OR "n6 fat*" OR "n 6 oil*" OR "n6 oil*" OR "omega 6" OR omega6):ti,ab,kw
73,596 results
#3 #1 AND #2
477 trials (final results)
4 reviews (final results)

SI2:

Table S1: Supplement complex information

Study	Supplement complex
Allegrini D et al., 2021<br>Italy	150mg of curcumin (curcuma longa l., rizoma) dry extract with minimum 95% of curcuminoids, AREDS2 components, 4mg astaxanthin (from haematococcus pluvialis flotow algae), and 20mg resveratrol (from roots of polygonum cuspidatum siebold)
Berrow EJ et al., 2013<br>UK	once per day<br>**Ocuvite Duo®** (Bausch and Lomb, Canada)<br>(Vitamin C 150 mg, Cupric oxide 400 µg, Vitamin E 15 mg (DL-a-tocopherol), Zinc oxide 20 mg, Lutein 12 mg, Zeaxanthin 0.6 mg, EPA 240 mg, DHA 840 mg)
Dawczynski J et al., 2013<br>Germany	I1: once per day **FloraGLO® Lutein®** (Lutein, Kemin Food L.C, Des Moines, IA)<br>(10 mg Lutein, 1 mg Z, 255 mg concentrated fish oil (there of 100 mg DHA, 30 mg EPA) and antioxidants (60 mg vitamin C, 20 mg vitamin E, 10 mg zinc, 0,25 mg copper))<br>I2: 20 mg Lutein, 2 mg Zeaxanthin Z, 500 mg concentrated fish oil (thereof 200 mg DHA, 60 mg EPA) and antioxidants (120 mg vitamin C, 40 mg vitamin E, 20 mg zinc, 0,5 mg copper) aka two tablets
Parravano M et al., 2019<br>Italy	Twice per day **Macuprev** ® (Farmaplus Italia s.r.l., Italy)<br>in total: lutein (20 mg), zeaxanthin (4 mg), N-acetylcysteine (140 mg), bromelain 2500GDU (80 mg), vitamin B12 (18 mg), vitamin D3 (800 IU), alphalipoic acid (140 mg), rutin (157 mg),<br>vitamin C (160 mg), zinc oxide (16 mg), Vaccinium myrtillus 36% anthocyanosides (90 mg), Ganoderma lucidum (600 mg)
Richer SP et al., 2004<br>USA	I1: 10mg lutein **FloraGlo® (** Kemin Foods International, Des Moines, Iowa)<br>I2: 10mg lutein (FLoraGLo) plus 2,500 IU vitamin A, 15,000 IU natural beta carotene (Betatenem®);<br>1,500-mg vitamin C (as calcium ascorbate-Ester CB®); 400 IU vitamin D3; 500 IU natural vitamin E (d-alpha tocopherol succinate); 50-mg vitamin B1; 10-mg vitamin B2; 70-mg vitamin B3; 50-mg vitamin B5; 50-mg vitamin B6; 500-mcg vitamin B12; 800-mcg folic acid; 300-mcg biotin; 500-mg calcium; 300-mg magnesium; 75-mcg iodine; 25-mg zinc (as zinc L-methionine-L-OptiZincB®); 1-mg copper; 2-mg manganese; 200-mcg selenium; 200-mcg chromium; 75-mcg molybdenum; 600-mcg lycopene; 160-mg bilberry extract (standardized to 25% anthocyanosides); 150-mg alpha lipoic acid; 200-mg N-acetyl cysteine; 100-mg quercetin; 100-mg rutin; 250-mg citrus bioflavonoids; 50-mg plant enzymes; 5-mg black pepper extract (BioperineB®); 325-mg malic acid;<br>900-mg taurine; 100-mg L-glycine; 10-mg L-glutathione; and 2-mg boron antioxidants and nutrients (**OcuPower**, Nutraceutical Sciences Institute (NSI) , Boynton Beach, Florida),
